# Food neophobia and its association with dietary choices and willingness to eat insects

**DOI:** 10.3389/fnut.2023.1150789

**Published:** 2023-07-12

**Authors:** Indee Hopkins, Asgar Farahnaky, Harsharn Gill, Jessica Danaher, Lisa P. Newman

**Affiliations:** ^1^DINE Lab, School of Science, STEM College, RMIT University, Melbourne, VIC, Australia; ^2^School of Science, STEM College, RMIT University, Melbourne, VIC, Australia

**Keywords:** food neophobia, entomophagy, edible insects, dietary choice, novel foods

## Abstract

Growing populations, changing dietary preferences and limitations on natural resources have meant that finding an alternative to traditional animal-based protein sources is a priority. Insects have been proposed as a possible solution due to their many benefits including low resource inputs and rich nutritional profile. However, insects are not consumed on a large scale by Australians. Food neophobia (reluctance to try new foods) could be contributing to this delay and as such, this study aimed to explore the role of food neophobia on protein food source habits and willingness to eat insects as food. A total of 601 participants (76.2% female, 23.8% male) completed an online survey which included a questionnaire measuring food neophobia status, participants’ self-reported usual protein dietary habits, their previous insect-eating experience, future willingness to eat insects, and potential motivations to include insects in their diet. Results indicated a strong association between food neophobia and participants’ dietary choices such as following a vegan or vegetarian diet (*p* = 0.024). In addition, food neophobia was correlated with a reduced likelihood of previous insect-eating experience (*p* < 0.001), as well as a decreased willingness to eat insects in the future (*p* < 0.001). This study provides a greater understanding of the role of food neophobia status and dietary choices in consumers’ willingness to eat insects and identifies possible motivating factors that may increase the likelihood of consumers’ future insect eating.

## Introduction

A combination of an expanding global population and increased *per capita* calorie intake (1) has meant that food production will need to increase by 70% by 2050 (2), with animal-based protein a priority (3). Constrained by limitations on the finite resources required for animal-based food production, finding an alternative solution is paramount. Entomophagy (eating insects) is a viable solution with many environmental benefits, including reduced land, water, and energy input requirements (4), as well as decreased greenhouse gas emissions (GHG) and thus a reduced exacerbation of climate change (5).

Insects have a high nutritional value, including protein (essential amino acids), polyunsaturated fatty acids such as omega-3 fatty acids, and micronutrients such as iron and calcium (6). Despite their reported environmental benefits, nutritional adequacy, and support from governing agencies such as the Food and Agriculture Organization of the United Nations (FAO) (4), the adoption of insects as a food source in Australia is still not widespread (7–9). Potential barriers to entomophagy include cultural and societal norms (10), unfavourable sensory appeal (11) and food neophobia (12). Furthermore, previous literature investigating Australian’s barriers to previous insect-eating experiences reported a lack of opportunity to try eating insects as the primary barrier, followed by disgust (13).

Food neophobia is the fear or aversion to new food items, and as such a decreased willingness to accept new food into one’s diet (14). While food neophobia is considered a personal characteristic it may be influenced by other factors such as a person’s culture, environmental factors, or socioeconomic circumstances which may restrict a person from trying or having exposure to unfamiliar or novel foods (15–18). Predominantly affecting children, food neophobia is thought to be an evolutionary function to prevent ingesting dangerous or poisonous foods and begins to subside during adolescents (19). Food neophobia has been associated with decreased diet quality and adverse health outcomes, such as an increased risk of type 2 diabetes (20) and increased body mass index (BMI) (21).

Previous literature asserts that low socioeconomic status may contribute to the development of food neophobia due to the limited opportunities for exposure to new or novel foods (22). It is well established that repeated exposure to a type of food increases the level of familiarity with the product and in turn, is associated with a greater level of acceptance. The lack of exposure to novel food products can lead to a lack of familiarity and increased levels of food neophobia (23). Low socioeconomic background may also result in the continued consumption of foods that are familiar rather than risk financial loss from trying a novel food product and not liking the product (24).

Previous research exploring protein consumption habits and willingness to eat insects is minimal. Although a recent publication on Australian consumers’ willingness to eat insects included additional findings regarding participants’ protein consumption habits, an association analysis between the two factors has not been explored (9). In a broader sense, a review on consumer acceptance of alternative proteins concluded that lifestyle significantly influenced consumers’ approval, with high meat consumption associated with increased receptiveness to cultured meats but decreased acceptance of plant-based meat alternatives such as algae and pulses (25).

While on a global level the available literature on the role of food neophobia in the acceptance of entomophagy has increased recently, the bulk of this has been from Europe where the concept of eating insects is new, unlike in Australia (26–28). Australians are often exposed to the concept of insects as food through formal education of our First Nations People and may have a certain level of familiarity that is lacking in European countries. Previous literature on the role of food neophobia and entomophagy is limited in the Australian context (7, 9) as is the availability of literature exploring possible associations between Australians’ dietary choices and a willingness to eat insects (9). As such, it was the objective of this study to evaluate the prevalence of food neophobia in an Australian adult sample and explore any associations between a participant’s food neophobia status, dietary choices, and their willingness to eat insects.

## Methodology

### National survey

An online survey investigating Australians’ food neophobia status and willingness towards adopting entomophagy was conducted with data collected between October 2019 and ceasing May 2020. The survey questions that formed the basis of this study’s investigation were part of a larger study titled ‘Australian’s awareness, perceptions, and willingness in the adoption of entomophagy (eating insects)’ and the survey was administered using Qualtrics, LLC (Qualtrics, SAP America Inc.). The project was approved by the RMIT College Human Ethics Advisory Network (CHEAN) project reference 2020-23026-10535.

Survey participant recruitment was via social media platforms (Facebook), with promotion on the researchers’ private accounts and manually posting study information in various community groups. Word-of-mouth recruitment was also used, with survey participants encouraged to share the survey within their networks. Participants were first screened to ensure they met the study’s inclusion criteria, which required participants to be aged ≥18 years and either Australian citizens or permanent residents. Participants were required to provide informed consent before the commencement of the survey, the duration of the survey was 10–15 min to avoid respondent fatigue (29). A full list of the survey questions related to this segment of the survey can be found in Supplementary information 1.

Question themes included in the survey were demographic (*What is the highest qualification you have completed?*), entomophagy experience (*Have you ever willingly eaten insects?*), and usual protein food source consumption habits (*How often do you eat any of the following? - Red meat (beef, lamb) etc.*). The Food Neophobia Scale (FNS) questionnaire was also included (14). The FNS scale is a validated tool (30, 31) and has become the standardized method for measuring levels of food neophobia (32). While there are alternative methods of calculating scores and determining classification using the FNS (33), the method used in this study is considered the standard methodology (14) and has been used in other studies (30, 34).

The FNS comprises 10 items, five food neophiliac (I will eat almost anything) and five food neophobic (I am very particular about the foods I will eat) statements. These items were evaluated on a 7-point Likert scale ranging from 1 = ‘I strongly disagree’ to 7 *=* ‘I strongly agree’ for neophiliac items and the reverse 1 = ‘I strongly agree’ to 7 = ‘I strongly disagree’ for neophobic items (Supplementary information 2). Participants were classified as either food neophiliac (tending to seek something new to taste), food neutral or food neophobic (having an aversion to trying new foods) (14).

Participants’ FNS scores have a possible range of between 10 to 70 (arbitrary unit), with FNS ranges obtained using the FNS mean scores from the study, plus or minus one standard deviation. For this study, the mean and standard deviation was 27.63 ± 11.33 and as such participants were classified as food neophiliac with a total score of ≤16, as food neutral with scores between 17 and 38, or as food neophobic with scores of ≥39 (14).

Participants were asked if they identified as either vegan, vegetarian, pescatarian, flexitarian, or none of the above. Notably, the dietary choice vegetarian was only stated and not specifically defined in this survey, meaning the inclusion and exclusion of various animal food sources in the diet were open to individual respondent interpretation. An example of this would be the individual interpretation of the term vegetarian which for some includes the consumption of white meats (fish, chicken) and for others, it excludes all meats including white meat (35).

The protein food source habits of participants were collected using a 6-point Likert scale. Participants were required to indicate the frequency of their consumption of a variety of food protein sources such as red meat (beef, lamb), seafood (fish, shellfish), and wild game (kangaroo, deer). Participants’ previous entomophagy experience data was collected using the open-ended question *‘Have you ever willingly eaten insects?’* and ‘*If yes, what type of insects have you eaten before?’*. The check-all-that-apply question *‘Would any of the following increase your willingness to eat insects or insect-based products?’* was utilised to explore possible motivations to increase the likelihood of future insect-based product consumption.

### Data analysis

To ensure the maximum amount of useful data and reduce the violation of the Chi-square assumptions, participants’ responses to the question *What is the highest qualification you have completed?* had the response categories ‘no formal education’, ‘≤Year 10 (Australian secondary school metric)’ and ‘≤Year 12 (Australian secondary school metric)’ combined into a single category ‘≤Year 12 (Australian secondary school metric)’ and the response categories ‘Trade/apprenticeship (Hairdresser, chef)‘and ‘Certificate/diploma (Childcare, technician)’ were combined into the category ‘Trade/Certificate’. Responses to the question *What is your total household income before tax?* Had the response categories ‘no income per week’, ‘$1–$119 per week’, ‘$120–$299 per week’ and ‘$300–$499 per week’ were combined into the one category ‘$0–$499 per week’. Similarly, the response categories ‘$500–$699 per week’ and ‘$700–$999 per week’ were combined into the one category ‘$500–$999 per week’ and responses ‘Do not know’ and ‘Do not want to answer’ were combined into the category ‘Unknown’. Additionally, participant responses to the question *If given the opportunity, how likely would it be for you to eat insects?* were recategorized from the separate categories ‘extremely likely’ and ‘somewhat likely’ into a single category ‘likely’. Similarly, the separate categories ‘extremely unlikely’ and ‘somewhat unlikely’ were combined into the single category ‘unlikely’. Response data were categorised and analysed using IBM SPSS 28 (Armonk, NY: IBM Corp).

Response data were analysed using descriptive techniques including frequencies and percentages. The Chi-square test was used to test for the presence of a relationship between the study’s nominal variables, with results presented as *χ^2^* (degrees of freedom, N = sample size) chi-square statistic value, and *value of p*. In situations when the assumptions for the Chi-square test were not met such as when >20% of the table’s cells had expected frequencies of <5 (36), a Spearman’s rank correlation coefficient was performed. Fisher’s exact test was performed when the Chi-square test and Spearman’s rank correlation coefficient were not appropriate. A significance level of *p* < 0.05 was applied for all statistical tests.

## Results

### Survey participants demographics

A total of 601 participants completed the survey (Table 1). Of these, 76.2% were female and 23.8% male, with the majority in age groups 25–34 years (31.4%) and 35–44 years (26.8%). More than half of the participants (59.1%) indicated their highest level of qualification attained was a university (tertiary) degree and just 16.0% reported ≤Year 12 (Australian secondary school metric). The household weekly income of participants as displayed in Table 1, highlights half of participants (50.4%) reported a ≥ $1,500 weekly income, followed by 17.0% of participants who reported $500–$999 per week. A segment of participants (11.8%) chose not to disclose household income information.

**Table 1:** Demographics of participants.

Demographics	Participants (*n*)	Percentage (%)
*Within gender*		
Male	143	23.8
Female	458	76.2
*Within age group (years)*		
18–24	56	9.3
25–34	189	31.4
35–44	161	26.8
45–54	101	16.8
55–64	73	12.1
≥65	21	3.5
*Within highest qualification*		
≤Year 12	96	16.0
Trade/certificate	150	25.0
University degree	355	59.1
*Within household weekly income (AUD)*		
$0–$499	39	6.5
$500–$999	102	17.0
$1,000–$1,499	86	14.3
≥$1,500	303	50.4
Unknown	71	11.8

### Participant food neophobia status

Participants’ level of food neophobia was evaluated with results indicating that 17.8% of participants (*n* = 107/601) were classified as food neophiliacs, 64.7% of participants (*n* = 389/601) were classified as food neutral and 17.5% of participants (*n* = 105/601) were classified as food neophobic (Table 2).

**Table 2:** Association between participant demographics and food neophobia status.

Food neophobia	Food neophiliac	Food neutral	Food neophobic	value of *p*
*% Within total participants*				
Total 601	17.8	64.7	17.5	
*% Within gender*				
Male	26.6	60.8	12.6	0.004^*^
Female	15.1	65.9	19.0	
*% Within age group (years)*				
18–24	12.5	57.1	30.4	0.024^*^
25–34	16.4	68.8	14.8	
35–44	23.0	61.5	15.5	
45–54	23.8	59.4	16.8	
55–64	11.0	69.9	19.2	
≥65	0.0	81.0	19.0	
*% Within highest qualification*				
≤Year 12	10.4	61.5	28.1	0.002^*^
Trade/certificate	14.7	64.7	20.1	
University degree	21.1	65.6	13.2	
*% With household weekly income (AUD)*				
$0–$499	17.9	61.5	20.5	0.716
$500–$999	15.7	67.6	16.7	
$1,000–$1,499	23.3	60.5	16.3	
≥$1,500	16.8	67.0	16.2	
Unknown	18.3	57.7	23.9	

Data presented as a percentage; Chi-squared test used for all analysis in the table, ^*^Significance indicated at *p* < 0.05.

There was a significant association observed between food neophobia status and participant’s gender (*χ*^2^ (2, *N* = 601) =11.105, *p* = 0.024), with females (19.0%, *n* = 87/458) more likely to exhibit food neophobia compared to males (12.6%, *n* = 18/143). A relationship was also observed between participants food neophobia status and age group (*χ*^2^ (10, *N* = 601) =20.633, *p* = 0.024), with the 18–24 age group (30.4%, *n* = 17/56) more likely to exhibit food neophobia compared to the 25–34 age group (14.8%, *n* = 28/189).

A further relationship was observed between a participant’s qualifications and their food neophobia status (*χ*^2^ (4, *N* = 601) = 16.929, *p* = 0.002), with participants who reported the highest qualification of ≤Year 12 more likely to be classified as food neophobic (28.1%, *n* = 27/96) than participants reporting a university degree (13.2%, *n* = 47/355).

No significant associations were observed between food neophobia status and participant’s household weekly income and food neophobia status (*χ*^2^ (8, *N* = 601) = 5.387, *p* = 0.716).

### Food neophobia status and dietary choices

Participants were questioned as to whether they choose to adhere to any dietary choice such as vegan or vegetarian (Table 3), of which, 77.9% (*n* = 468) indicated they did not follow specific dietary regime. From the 22.1% (*n* = 133) of participants that report adherence to a dietary regime, flexitarian was the highest selected dietary choice at 10.0% (*n* = 60/601) and the least selected dietary choice was pescatarian at 2.8% (*n* = 17/601).

**Table 3:** Association between participant demographics, food neophobia status and dietary choice.

Dietary choice	Vegan	Vegetarian	Pescatarian	Flexitarian	None	Value of *p*
*% Within total participants*						
Total 601	3.0	6.3	2.8	10.0	77.9	
*% Within food neophobia status*						
Food neophiliac	0.9	0.9	0.9	15.0	82.2	0.024^^,*^
Food neutral	3.6	7.5	3.3	10.0	75.6	
Food neophobic	2.9	7.6	2.9	4.8	81.9	
*% Within gender*						
Male	2.8	4.9	0.7	6.3	85.3	0.110
Female	3.1	6.8	3.5	11.1	75.5	
*% Within age group (years)*						
18–24	5.4	7.1	1.8	7.1	78.6	0.768^#^
25–34	0.5	12.2	2.6	10.6	74.1	
35–44	5.0	3.7	2.5	9.9	78.9	
45–54	2.0	5.0	2.0	9.9	81.2	
55–64	5.5	0.0	6.8	8.2	79.5	
≥65	0.0	0.0	0.0	19.0	81.0	
*% Within highest qualification*						
≤Year 12	1.0	2.1	2.1	7.3	87.5	0.169^^^
Trade/certificate	2.0	4.7	2.0	9.3	82.0	
University degree	3.9	8.2	3.4	11.0	73.5	
*% With household weekly income (AUD)*						
$0–$499	2.6	7.7	0.0	10.3	79.5	0.454^#^
$500–$999	2.9	8.8	2.0	12.7	73.5	
$1,000–$1,499	1.2	9.3	0.0	9.3	80.2	
≥$1,500	3.3	4.6	3.3	8.9	79.9	
Unknown	4.2	5.6	7.0	11.3	71.8	

Data presented as a percentage, Chi-squared analysis used unless indicated, ^^^Fisher’s exact test, ^*^Significance indicated at *p* < 0.05, ^#^Value of *p* presented from the Chi-squared analysis between adherence to a dietary choice (yes or no) and household weekly income.

An association was detected between participant’s dietary choice and food neophobia status (two-tailed, *p* = 0.024), with food neophiliac participants more likely to adhere to the dietary choice flexitarian (15.0%, *n* = 16/107) than food neophobic participants (4.8%, *n* = 5/105). However, food neophobic participants are more likely to adhere to the dietary choice vegan (2.9%, *n* = 3/105), vegetarian (7.6%, *n* = 8/105) and pescatarian (2.9%, *n* = 3/105) than food neophiliac participants (vegan 0.9%, *n* = 1/10, vegetarian 0.9%, *n* = 1/10, pescatarian 0.9%, *n* = 1/10).

No associations were detected between participant’s dietary choice and their gender (*χ*^2^ (4, *N* = 601) = 7.532, *p* = 0.110) or between adherence to a dietary choice and participant’s age group (*χ*^2^ (5, *N* = 601) = 2.559, *p* = 0.768). No association was observed between participants highest qualification (two-tailed, *p* = 0.169) or household weekly income (*χ*^2^ (4, *N* = 601) = 3.657, *p* = 0.454).

### Protein food source habits

Of the protein sources listed, 58.2% (*n* = 350/601) of participants reported they consumed dairy (milk, yoghurt) daily, this was followed by 18.0% (*n* = 108/601) of participants consuming plant-based protein foods (legumes, tofu) daily. The least consumed protein source was wild game (kangaroo, deer) with 63.4% (*n* = 381/601) of participants reporting as never eaten, followed by white meat (pork) with 22.8% (*n* = 137/601) of participants reporting they never consume this protein source (Table 4).

**Table 4:** Participant protein food source habits (*N* = 601).

Protein food source	Consumption frequency					
	Daily	3+ days a week	1–2 days a week	Less than weekly	1–2 days a month	Never
Red meat (beef, lamb)	1.3	23.5	44.8	10.3	5.2	15.0
White meat (pork)	0.5	4.3	22.5	31.8	18.1	22.8
White meat (poultry)	1.8	31.1	42.8	8.8	3.3	12.1
Seafood (fish, shellfish)	0.3	3.7	28.5	32.4	18.8	16.3
Wild game (kangaroo, deer)	0.2	1.3	2.3	12.8	20.0	63.4
Dairy (milk, yoghurt)	58.2	20.8	10.1	3.8	1.5	5.5
Eggs	12.5	29.1	33.8	13.1	5.3	6.2
Plant-based protein foods (legumes, tofu)	18.0	23.3	24.3	17.0	8.3	9.2

Data presented as percentage.

An association was detected between food neophobia status and red meat (beef, lamb) consumption (*χ*^2^ (10, *N* = 601) = 25.698, *p* = 0.004), white meat (poultry) (*r*_s_ [601] = 0.108, *p* = 0.008), seafood (fish, shellfish) (*r*_s_ [601] = 0.205, *p* < 0.001), wild game (kangaroo, deer) (*r*_s_ [601] = 0.177, *p* < 0.001), eggs (*χ*^2^ (10, *N* = 601) = 19.058, *p* = 0.040) and plant-based protein foods (legumes, tofu) (*χ*^2^ (10, *N* = 601) = 50.664, *p* < 0.001). Food neophobic participants were more likely to report never consuming red meat (beef, lamb), white meat (poultry), seafood (fish, shellfish), wild game (kangaroo, deer) and plant-based protein foods (legumes, tofu), compared to food neophiliac participants (Table 5).

**Table 5:** Association between participant food neophobia status and protein food source habits (*N* = 601).

Protein food source	Food neophobia
Red meat (beef, lamb)	0.004^*^
White meat (pork)	0.178^~^
White meat (poultry)	0.008^~,*^
Seafood (fish, shellfish)	<0.001^~,*^
Wild game (kangaroo, deer)	<0.001^~,*^
Dairy (milk, yoghurt)	0.165^~^
Eggs	0.040^*^
Plant-based protein foods (legumes, tofu)	<0.001^*^

Chi-squared analysis used unless indicated, ^~^Spearman’s rank correlation coefficient, ^*^Significance indicated at *p* < 0.05.

No associations were identified between food neophobia status and white meat (pork) (*r*_s_ [601] = 0.055, *p* = 0.178), and dairy (milk, yoghurt) (*r*_s_ [601] = 0.057, *p* = 0.156).

### Previous insect eating experience

From 601 participants, 35.4% (*n* = 213) reported they had previously eaten insects. An association was identified between participants level of food neophobia and the likelihood of them previously eating insects (*χ*^2^ (2, *N* = 601) = 48.084, *p* < 0.001), with food neophobic participants (12.4%, *n* = 13/105) less likely to have previously eaten insects compared to food neophiliac participants (57.9%, *n* = 62/107) (Table 6). Participants’ dietary choices were associated with the likelihood of reporting previous insect-eating experiences (*χ*^2^ (4, *N* = 601) = 15.394, *p* = 0.004), with participants following a flexitarian diet significantly more likely to have previously tried eating insects (51.7%, *n* = 31/60) than vegans (5.6%, *n* = 1/18), vegetarians (39.5%, *n* = 15/38), and pescatarians (23.5%, *n* = 4/17).

**Table 6:** Association between participants’ food neophobia status, dietary choice, and previous insect eating experience.

Have you eaten insects before?	Yes	No	Value of *p*
*% Within total participants*			
Total 601	35.4	64.6	
*% Within food neophobia status*			
Food neophiliac	57.9	42.1	<0.001^*^
Food neutral	35.5	64.5	
Food neophobic	12.4	87.6	
*% Within dietary choice*			
Vegan	5.6	94.4	0.004^*^
Vegetarian	39.5	60.5	
Pescatarian	23.5	76.5	
Flexitarian	51.7	48.3	
None of the above	34.6	65.4	
*% Within highest qualification*			
≤Year 12	24.0	76.0	<0.001^*^
Trade/certificate	26.0	74.0	
University degree	42.5	57.5	
*% With household weekly income (AUD)*			
$0–$499	33.3	66.7	0.714
$500–$999	36.3	63.7	
$1,000–$1,499	32.6	67.4	
≥$1,500	37.6	62.4	
Unknown	29.6	70.4	

Data presented as a percentage, Chi-squared test used for all analysis in the table, ^*^Significance indicated at *p* < 0.05.

Furthermore, participants’ highest qualifications were associated with the likelihood of reporting previous insect-eating experience (*χ*^2^ (2, *N* = 601) = 19.184, *p* < 0.001), with participants reporting a university degree (42.5%, *n* = 151/355) significantly more likely to report having previous insect-eating experience compared to participants reporting the highest qualification of ≤Year 12 (24.0%, *n* = 23/96).

No association was observed between participants’ household weekly income and their likelihood of reporting previous insect-eating experiences (*χ*^2^ (4, *N* = 601) = 2.117, *p* = 0.714).

### Future likelihood to eat insects

The future likelihood of participants to eat insects (*N* = 601) was evaluated (Table 7), where 56.2% (*n* = 338) of participants reported they would be ‘likely’, 10.1% (*n* = 61) would be ‘neither likely nor unlikely’ and 33.6% (*n* = 202) of participants reported they would be ‘unlikely’ to consume insects in the future.

**Table 7:** Association between food neophobia status, dietary choice, and likelihood to eat insects in the future.

Likelihood to eat insects	Likely	Neither likely nor unlikely	Unlikely	Value of *p*
*% Within total participants*				
Total 601	56.2	10.1	33.6	
*% Within food neophobia status*				
Food neophiliac	26.3	11.5	4.5	<0.001^*^
Food neutral	65.7	68.9	61.9	
Food neophobic	8.0	19.7	33.7	
*% Within dietary choice*				
Vegan	11.1	5.6	83.3	<0.001^*^
Vegetarian	31.6	13.2	55.3	
Pescatarian	41.2	17.6	41.2	
Flexitarian	70.0	10.0	20.0	
None of the above	58.8	9.8	31.4	
*% Within highest qualification*				
≤Year 12	42.7	14.6	42.7	0.057
Trade/certificate	58.0	10.7	31.3	
University degree	59.2	8.7	32.1	
*% With household weekly income (AUD)*				
$0–$499	51.3	7.7	41.0	0.221
$500–$999	57.8	9.8	32.4	
$1,000–$1,499	67.4	7.0	25.6	
≥$1,500	56.1	10.2	33.7	
Unknown	43.7	15.5	40.8	

Data presented as a percentage, Chi-squared test used for all analysis in the table, ^*^Significance indicated at *p* < 0.05.

A strong association between food neophobia status and the likelihood of future insect consumption was observed (*χ*^2^ (4, *N* = 601) = 124.232, *p* < 0.001), with food neophiliac participants (88.8%, *n* = 95/107) significantly more likely to eat insects in the future compared to food neophobic participants (25.2%, *n* = 27/107).

An association was also observed between participants’ dietary choice and likelihood to eat insects in the future (*χ*^2^ (8, *N* = 601) = 38.377, *p* < 0.001). Participants reporting adherence to a flexitarian diet were more likely to report they would be likely to eat insects in the future (70.0%, *n* = 42/60) than dietary choices vegan (11.1%, *n* = 2/18), vegetarian (31.6%, *n* = 12/38), and pescatarian (41.2%, *n* = 7/17) (Table 7).

No association was observed between highest qualification (*χ*^2^ (4, *N* = 601) = 9.178, *p* = 0.057), or participants’ household weekly income and likelihood to eat insects in the future (*χ*^2^ (8, *N* = 601) = 10.627, *p* = 0.221).

### Motivating factors to increase future willingness to eat insects

Participants were questioned on the efficacy of various motivating factors that may increase future willingness to eat insects (Table 8). Of the 601 participants, 22.6% (*n* = 136) reported that they would be *‘*unwilling to consume insects’. From the remaining participants (*n* = 465), the main motivating factors reported were ‘greater knowledge of the nutritional benefits of insects’ and ‘improved access to insect products’ equally at 73.1% (*n* = 340/465).

**Table 8:** Motivating factors that may increase the likelihood of participants eating insects in the future (*n* = 465).

Motivating factor	Yes	No
Increased knowledge regarding nutritional benefits	73.1	26.9
Increased accessibility to insect products	73.1	26.9
Endorsement of safety from a governing body	62.4	37.6
Regular appearance in mainstream media	58.9	41.1
Designated production facilities	41.7	58.3
Increased environmental burden from animal protein	38.5	61.5
Other	11.2	88.8

Data presented as a percentage; Values do not add up to 100% because participants were directed to check-all-that-apply.

A further 11.2% (*n* = 52/465) of participants, using an open-ended question provided ‘other’ factors that may increase their willingness to consume insects. Other factors reported by neophiliac participants included ‘reduced cost in comparison to other options’ and ‘further environmental impact assessments on largescale production’. Factors reported by food neophobic participants included ‘try before you buy promotions’ and ‘seeing other people eating them regularly’. Both food neophiliac and food neophobic participants reported ‘Improvements in product presentation (no identifiable insect characteristics)’ (Supplementary Table S2).

An association between food neophobia status and the motivating factor ‘Increased accessibility to insect products’ was observed (*χ*^2^ (2, *n* = 465) = 26.464, *p* < 0.001), with food neophiliac participants (81.6%, *n* = 84/103) significantly more likely to report ‘Increased accessibility to insect products’ compared to both food neutral (75.2%, *n* = 233/310) and food neophobic participants (44.2%, *n* = 23/52) as a motivating factor (Table 9).

**Table 9:** Association between food neophobia status and motivating factors that may increase the likelihood of eating insects (*n* = 465).

Motivating factor	Food neophiliac		Food neutral		Food neophobic		value of *p*
	Yes	No	Yes	No	Yes	No	
Increased knowledge regarding nutritional benefits	71.8	28.2	74.5	25.5	67.3	32.7	0.526
Increased accessibility to insect products	81.6	18.4	75.2	24.8	44.2	55.8	<0.001^*^
Endorsement of safety from a governing body	62.1	37.9	62.9	37.1	59.6	40.4	0.901
Regular appearance in mainstream media	54.4	45.6	63.9	36.1	38.5	61.5	0.001^*^
Designated production facilities	49.5	50.5	41.9	58.1	25.0	75.0	0.014^*^
Increased environmental burden from animal protein	46.6	53.4	38.7	61.3	21.2	78.8	0.009^*^
Other	10.7	89.3	10.0	90.0	19.2	80.8	0.146

Data presented as a percentage, values do not add up to 100% because participants were directed to check-all-that-apply, Chi-squared test used for all analysis in the table, ^*^Significance indicated at *p* < 0.05.

An association between food neophobia status and the motivating factor ‘Designated production facilities’ was identified (*χ*^2^ (2, *n* = 465) = 8.558, *p* = 0.014), with food neophobic participants (75.0%, *n* = 39/52) more likely to indicate that ‘Designated production facilities’ would not be a motivating factor to increase future willingness to eat insects compared to both food neutral (58.1%, *n* = 180/310) and food neophiliac participants (50.5%, *n* = 52/103).

An association was identified between participants food neophobia status and the motivating factor ‘Regular appearance in mainstream media’ (*χ*^2^ (2, *n* = 465) = 13.013, *p* = 0.001), with and food neophobic participants (61.5%, *n* = 32/52) more likely to report ‘Regular appearance in mainstream media’ would not be a motivating factor to increase future willingness to eat insects compared to both food neutral (36.1%, *n* = 112/310) and food neophiliac participants (45.6%, *n* = 47/103).

A further association was observed between food neophobia status and the motivating factor ‘Increased environmental burden from animal protein’ (*χ*^2^ (2, *n* = 465) =9.470, *p* = 0.009), with food neophiliac participants (46.6%, *n* = 48/103) more likely to report ‘Increased environmental burden from animal protein’ compared to both food neutral (38.7%, *n* = 120/310) and food neophobic participants (21.2%, *n* = 11/52) as a potential motivation to increase future willingness to eat insects.

No association was observed between participant food neophobia status and the motivating factors included ‘Increased knowledge regarding nutritional benefits’ (*χ*^2^ (2, *n* = 465) = 1.286, *p* = 0.526), ‘Endorsement of safety from a governing body’ (*χ*^2^ (2, *n* = 465) = 0.208, *p* = 0.901), and ‘Other’ (*χ*^2^ (2, *n* = 465) = 3.854, *p* = 0.146).

## Discussion

The findings of this study highlight that food neophobia status is correlated with past insect-eating experience, with food neophobic individuals displaying a reduced likelihood of previously trying insects when compared to their food neophiliac counterparts. It is also evident that food neophobia status is correlated with future willingness to eat insects, with individuals classified as food neophobic reporting a reduced willingness to eat insects compared to those classified as food neutral or food neophiliac. The results also showed that food neophobia status influences dietary lifestyle, with food neophiliac individuals more likely to adhere to a flexitarian dietary choice when compared food neophobic individuals, who were more likely to adhere to vegan, vegetarian or pescatarian dietary choice.

These results are similar to previous research reporting food neophobia status as a major predictor of an individual’s likelihood to eat insects (9, 37–39). In this study, 35.4% of the total participant group had previously eaten insects, with 57.9% of the food neophiliac participants reporting a previous insect-eating experience compared with just 12.4% of food neophobic participants. The prevalence of previous insect-eating experience amongst both food neophiliac and food neophobic individuals was higher in our study compared with similar Australian research, which reported 36.0% of food neophiliac and 11.0% of food neophobic participants having previous insect-eating experience (9). This difference may be attributed to the higher income level of participants in this study, with the majority (50.4%) reporting a household income in the highest response category of ≥$1,500 AUD weekly. We anticipate that due to a higher household income, participants may have a greater opportunity to travel, and as such more opportunity to eat insects, with previous research finding almost half (46.5%) of Australians’ entomophagy experiences have taken place while on holiday or abroad (13).

Furthermore, in this study, ‘Increased knowledge regarding nutritional benefits’ was the most marked motivator influencing food neophobic participants’ likelihood of trying edible insects (65.6%) and food neophiliac participants reported ‘Increased accessibility to insect products’ (81.6%). These results are not surprising, as food neophobia is a fear of trying new foods and as such providing increased information regarding insects is likely to increase familiarity with insects as food. Familiarity is a strong predictor of the acceptance of new or novel foods (40). Australia has seen a marked expansion in the ‘insects for food’ sector (27, 41) and the availability of information has been found to be effective in increasing the willingness to eat insects (39, 42, 43). Rumpold and Langen observed that by providing participants who were initially unwilling to taste insects with information regarding nutrition, safety, taste, trends, and history of edible insects, there was an increase of 19.2% in participants willing to taste insects (44).

We observed dietary choice to also be associated with the likelihood of participants reporting a future likelihood to eat insects, with participants adhering to a vegan diet choice being the most unwilling of participants. Similarly, a Finnish study (45) indicated that willingness to eat insects was lower in vegans compared to non-vegan vegetarians and omnivores. However, in Elorinne et al. willingness to eat insects was higher in the non-vegan vegetarian-based dietary group and not in participants who would be considered omnivores. This was not the case with our study as the omnivorous dietary choices flexitarian and pescatarian were more likely to indicate a willingness to eat insects than the vegetarian dietary group. This discrepancy may be due to the underrepresentation of participants in our study who identified as vegan, or vegetarian compared to the omnivorous dietary choices which could have resulted in decreased sensitivity to detect any noticeable association. Additionally, studies exploring dietary choices (i.e., pescatarian) and food neophobia are scarce and where available include populations of mostly females, aged ≤50 years (45, 46). The results of the current study showed a correlation between food neophobia status and dietary choice, with those identifying as vegetarians more likely to be food neophobic than those who adhered to the omnivorous diet choices pescatarian and flexitarian. This was in partial agreeance with Elorinne et al. which reported vegans and vegetarians were more food neophobic than omnivores. However, vegans in this study were less likely to be considered food neophobic than pescatarians. In contrast, a study from the United States (46) stated that the dietary choices of semi-vegetarians (those avoiding red meat, but including fish, poultry, and sometimes pork) and omnivores were associated with an increasing prevalence of food neophobia. These discrepancies may be due to different methodologies used by different studies. For example, both Elorinne et al. and Forestell et al. reported administering the Pliner and Hobden Food neophobia scale, however they did not include the methods of calculating and establishing the classification of participants as either food neophiliac, food neutral or food neophobic for their studies. As such the different methodologies used in these studies restrict the ability to draw any valid conclusions regarding any possible correlation between food neophobia status and dietary choices.

Furthermore, this study revealed that food neophobia was associated with decreased consumption of red meat (beef, lamb), white meat (poultry), seafood (fish, shellfish), wild game (kangaroo, deer), and plant-based protein foods (legumes, tofu), when compared to the consumption habits of food neophiliac individuals. However, there was no association observed between food neophobia status and white meat (pork), dairy (milk, yoghurt), and eggs. Similar observations have also been reported that increased food neophobia status was associated with a decreased consumption of fish (except white fish) and game meats but not with red meat and white meat (poultry) in Portuguese adults (47). Michel et al. also reported that participants with a higher food neophobia status were more frequent consumers of red meat (48). This discrepancy may be attributed to the proportion of male and female participants in the studies. Previous research has shown that red meat intake is higher in males than females across all age groups (49). Birrell et al. reported males aged between 2 years to 71 years had a higher intake of all meat types than females of the same age. The large female sample (76.2%) in our study could explain the decreased intake of red meat (beef, lamb), white meat (poultry), seafood (fish, shellfish), and wild game (kangaroo, deer) reported. Previous research has shown that people identifying as actively reducing their intake of fresh meat are more likely to report a willingness to eat insects as food (38).

A notable limitation of this study that needs to be taken into consideration when interpreting the results is that the majority of participants surveyed were aged 25–44 years (58.2%). Comparatively the age group ≥65 years was represented by just 3.5%. Literature exploring the role of age on willingness to eat insects reports conflicting assertions (50–52). Verbeke et al. reported a greater willingness in the younger generations to eat insects; every 10-year age increase was associated with a 27.0% reduction in participants’ willingness to accept insects as meat substitutes. Similarly, Wilkinson et al. also reported that an increase in age was associated with decreased acceptance of insects as food (9). However, Kane and Dermiki and Hartmann and Siegrist observed no such relationship between age and willingness to eat insects (50, 53). As such, the possibility of an age-related influence should be considered when interpreting the results of this study.

## Conclusion

In conclusion, this research indicated an association between food neophobia status, and level of future willingness to eat insects, with food neophobia associated with reduced likelihood to eat insects. Food neophobia status was not associated with a dietary choice, however, it was correlated with red meat (beef, lamb), white meat (poultry), seafood (fish, shellfish), wild game (kangaroo, deer) and plant-based protein foods (legumes, tofu) consumption frequency. The dietary choice was associated with all listed protein sources’ consumption frequency, previous insect-eating experience, and future willingness to eat insects. The results of this research indicate that to increase the likelihood of insects becoming a viable alternative to traditional protein sources, the primary requirement will be to develop strategies to overcome the barrier of food neophobia, such as educational campaigns regarding the benefits of insects as food.

Future research should continue examining the relationship between food neophobia and novel food acceptance, whilst developing strategies to increase consumer exposure to insect-based food to create product familiarity.

## Data availability statement

The raw data supporting the conclusions of this article will be made available by the authors, without undue reservation.

## Ethics statement

The studies involving human participants were reviewed and approved by RMIT College Human Ethics Advisory Network (CHEAN) project reference 2020-23026-10535. The patients/participants provided their written informed consent to participate in this study.

## Author contributions

IH, AF, HG, JD, and LN contributed to the development of the research protocol and provided the intellectual input to the final manuscript. IH completed the data collection, data analysis, and drafting of the paper. LN and JD monitored the data collection. IH and LN developed the statistical analysis plan. AF, HG, JD, and LN revised the draft paper. All authors contributed to the article and approved the submitted version.

## Conflict of interest

The authors declare that the research was conducted in the absence of any commercial or financial relationships that could be construed as a potential conflict of interest.

## Publisher’s note

All claims expressed in this article are solely those of the authors and do not necessarily represent those of their affiliated organizations, or those of the publisher, the editors and the reviewers. Any product that may be evaluated in this article, or claim that may be made by its manufacturer, is not guaranteed or endorsed by the publisher.

## Supplementary material

The Supplementary material for this article can be found online at: https://www.frontiersin.org/articles/10.3389/fnut.2023.1150789/full#supplementary-material
