# Supplementary material for: Food neophobia and its association with dietary choices and willingness to eat insects
Source: Front Nutr. 2023 Jul 12;10:1150789. doi: 10.3389/fnut.2023.1150789 (PMC10369065; doi:10.3389/fnut.2023.1150789)
Supplement: Supplementary file 1 [file Data_Sheet_1.docx]

Supplementary Material

**Food neophobia and its association with dietary choices and willingness to eat insects**

**Indee Hopkins^1^, Asgar Farahnaky^2^, Harsharn Gill^2^, Jessica Danaher^1^ and Lisa P Newman^1*^**

^1 DINE Lab, School of Science, STEM College, RMIT University, Melbourne, Australia^

^2 School of Science, STEM College, RMIT University, Melbourne, Australia^

*** Correspondence:**Dr Lisa Newman

Dine Lab, School of Science, STEM College

RMIT University, Bundoora West Campus

Victoria, 3083

Email: [lisa.newman@rmit.edu.au](mailto:lisa.newman@rmit.edu.au)

Ph: +61 (03) 9925 6113

**Questionnaire**

What is your citizenship status?

- Australian citizen
- Permanent resident
- Other/ non-Australian citizen

What is your age group?

- ≤17
- 18-24
- 25-34
- 35-44
- 45-54
- 55-64
- ≥65

What is your gender?

- Male
- Female

What is the HIGHEST qualification you have completed?

- No formal qualifications
- Year 10 or equivalent (School Certificate)
- Year 12 or equivalent (VCE, IB)
- Trade/apprenticeship (Hairdresser, chef)
- Certificate/diploma (Childcare, technician)
- University degree

What is your total household income before tax?

- No income
- $1-$119 per week ($1-$6,239 annually)
- $120-$299 per week ($6,240-$15,999 annually)
- $300-$499 per week ($16,000-$25,999 annually)
- $500-$699 per week ($26,000-$36,999 annually)
- $700-$999 per week ($37,000-$51,999 annually)
- $1,000-$1,499 per week ($52,000-$77,999 annually)
- $1,500 or more per week ($78,000 or more annually)
- Don't know
- Don't want to answer

How often do you eat any of the following?

|  | Daily | 3+ days a week | 1-2 days a week | Less than weekly | 1-2 days a month | Never |
| --- | --- | --- | --- | --- | --- | --- |
| Red meat (beef, lamb) |  |  |  |  |  |  |
| White meat (pork) |  |  |  |  |  |  |
| White meat (poultry) |  |  |  |  |  |  |
| Seafood (fish, shellfish) |  |  |  |  |  |  |
| Wild meat (kangaroo, deer) |  |  |  |  |  |  |
| Dairy (Milk, yoghurt, cheese) |  |  |  |  |  |  |
| Eggs |  |  |  |  |  |  |
| Plant-based proteins foods (legumes, tofu) |  |  |  |  |  |  |

Do you identify as

- Vegan
- Vegetarian
- Pescatarian
- Flexitarian
- None of the above

Have you ever willingly eaten insects?

- Yes
- No

If given the opportunity, how likely would it be for you to eat insects?

- Extremely likely
- Somewhat likely
- Neither likely nor unlikely
- Somewhat unlikely
- Extremely unlikely

**Supplementary Information 2**

**Food Neophobia Scale (FNS)** (Pliner & Hobden, 1992)

1. I am constantly sampling new and different foods.

2. I don’t trust new foods.

3. If I don’t know what is in a food, I won’t try it.

4. I like foods from different countries.

5. Ethnic food looks too weird to eat.

6. At dinner parties, I will try a new food.

7. I am afraid to eat things I have never had before.

8. I am very particular about the foods I will eat.

9. I will eat almost anything.

10. I like to try new ethnic restaurants.

**Supplementary Tables**

Supplementary Table 1. Descriptive statistics of the food neophobia scale results (n=601)

| Question | Mean (µ) | ± s. d |
| --- | --- | --- |
| I am constantly sampling new and different foods. (R) | 2.81 | 1.51 |
| I don’t trust new foods. | 2.71 | 1.48 |
| If I don’t know what is in a food, I won’t try it. | 3.86 | 1.94 |
| I like foods from different countries. (R) | 1.84 | 1.22 |
| Ethnic food looks too weird to eat. | 2.15 | 1.36 |
| At dinner parties, I will try a new food. (R) | 2.06 | 1.23 |
| I am afraid to eat things I have never had before. | 2.72 | 1.68 |
| I am very particular about the foods I will eat. | 3.63 | 1.88 |
| I will eat almost anything. (R) | 3.44 | 1.90 |
| I like to try new ethnic restaurants. (R) | 2.41 | 1.49 |

(R) question has been reversed coded.

Supplementary Table 2. Other motivating factors that may increase willingness to eat insects.

| Food neophiliac |
| --- |
| Reduced cost comparison to other options  Improvements in product presentation (no identifiable insect characteristics)  Further environmental impact assessments on largescale production  Knowledge of how to prepare  Taste |
| Food neophobic  Seeing other people eating them regularly  Starvation  Improvements in product presentation (no identifiable insect characteristics)  Try before you buy promotions |
